# Sotatercept for Connective Tissue Disease-Associated Pulmonary Arterial Hypertension with Concomitant Interstitial Lung Disease: Efficacy and Safety Insights

**DOI:** 10.3390/jcm14155177

**Published:** 2025-07-22

**Authors:** Chebly Dagher, Maria Akiki, Kristin Swanson, Brett Carollo, Garett Fiscus, Harrison W. Farber, Raj Parikh

**Affiliations:** 1 Department of Internal Medicine, University of Connecticut, Farmington, CT 06269, USA; 2 Division of Pulmonary, Critical Care and Sleep, Hartford Hospital, Hartford, CT 06106, USA; 3 Division of Pulmonary, Critical Care and Sleep, School of Medicine, University of Connecticut, Farmington, CT 06030, USA; 4 Division of Pulmonary, Sleep and Critical Care Medicine, Tufts Medical Center, Boston, MA 02111, USA

**Keywords:** pulmonary arterial hypertension, sotatercept, interstitial lung disease, connective tissue disease, group 1 pulmonary hypertension, group 3 pulmonary hypertension

## Abstract

**Background/Objectives**: Sotatercept has demonstrated efficacy in pulmonary arterial hypertension (PAH), but its use has not been studied in patients with Group 3 pulmonary hypertension (PH). Additionally, patients with connective tissue disease-associated PAH (CTD-PAH) were underrepresented in the STELLAR trial. Given the limited treatment options for pulmonary hypertension in patients with interstitial lung disease (PH-ILD), this study aimed to evaluate the use of sotatercept in CTD-PAH patients with concomitant ILD. **Methods**: Eligible patients (*n* = 7) had a confirmed diagnosis of CTD-PAH with concomitant ILD. The patients were already receiving background PAH therapy. Baseline hemodynamic and clinical measurements were reassessed after 24 weeks of sotatercept therapy. The variables assessed included six-minute walk distance (6MWD), pulmonary vascular resistance (PVR), echocardiographic right ventricular systolic pressure (eRVSP), N-terminal pro-brain natriuretic peptide (NT-proBNP) levels, World Health Organization (WHO) functional class, and supplemental oxygen requirements. **Results**: The study included seven patients with a mean age of 57 years (range: 39–73 years). After 24 weeks, the mean 6MWT distance increased from 211 m to 348 m (*p* < 0.01). Mean PVR decreased from 7.77 WU at baseline to 4.53 WU (*p* < 0.01). Mean eRVSP decreased from 79.43 mmHg to 54.14 mmHg (*p* < 0.01). NT-proBNP decreased from 3056.86 pg/mL to 1404.29 pg/mL (*p* < 0.01). The WHO functional class and supplemental oxygen requirements improved in all patients. **Conclusions**: Sotatercept was tolerated in patients with CTD-PAH and ILD, with no evidence of adverse respiratory effects. When added to foundational PAH therapy, sotatercept resulted in significant improvements across multiple parameters. These findings suggest that sotatercept may be a promising therapeutic option as an adjunctive treatment in this patient population.

## 1. Introduction

Pulmonary hypertension (PH) is a chronic, progressive cardiopulmonary disorder defined by a mean pulmonary artery pressure (mPAP) greater than 20 mmHg [1]. World Health Organization (WHO) Group 1 pulmonary arterial hypertension (PAH) is a sub-population of precapillary PH that may be idiopathic, heritable, drug-induced, or associated with conditions such as connective tissue disease (CTD) [2]. Meanwhile, WHO Group 3 PH is associated with lung diseases (LD), including obstructive and restrictive pulmonary disorders, such as interstitial lung disease (ILD) [2].

PAH is characterized by progressive vascular remodeling, leading to increased pulmonary pressures and right ventricular failure [3]. Current treatments focus on reducing vascular resistance through phosphodiesterase-5 inhibitors (PDE-5i), endothelin receptor antagonists (ERAs), soluble guanylate cyclase (sGC) stimulators, and prostacyclin pathway agents to improve vasodilation and slow disease progression [1,3]. Recently, sotatercept, an activin signaling inhibitor (ASI), received FDA approval for the treatment of PAH following the STELLAR trial [4]. By restoring the balance between pro- and anti-proliferative signaling in pulmonary vasculature, sotatercept has demonstrated clinical and hemodynamic improvements, even when added to traditional PAH-specific therapies [4]. CTD is the most common underlying condition associated with PAH, and its presence is linked to a worse prognosis compared to idiopathic PAH [5,6]. Despite the significant burden of CTD-PAH, patients with this condition were underrepresented in the STELLAR trial [4].

Managing CTD-PAH becomes significantly more challenging in the presence of ILD, as it exacerbates disease severity and complicates PAH-targeted therapy due to concerns about worsening ventilation-perfusion (V/Q) mismatch and potential harm [7,8,9,10]. This population requires careful treatment, balancing the benefits of PAH-directed therapies against the risks of worsening gas exchange [10]. Most randomized controlled trials (RCTs) evaluating PAH-specific therapies in PH-ILD have shown limited or no benefit, with some studies even reporting worsened outcomes [7,8,9,10]. The INCREASE trial, which evaluated the role of inhaled treprostinil in this population, remains the only RCT to demonstrate clinical and hemodynamic improvement in PH-ILD, highlighting the scarcity of effective treatment options in this population [11].

Given the limited efficacy and potential risks of traditional PAH therapies in patients with ILD, there is a critical need for novel treatments that target pulmonary vascular remodeling without exacerbating gas exchange abnormalities. To assess the potential of sotatercept in this population, we examined its efficacy, safety, and hemodynamic effects in a cohort of patients with CTD-PAH and concomitant ILD.

## 2. Materials and Methods

We retrospectively evaluated seven patients with PAH diagnosed using right heart catheterization (RHC) criteria for pre-capillary PH, defined as a mPAP > 20 mmHg, pulmonary capillary wedge pressure (PCWP) < 15 mmHg, and pulmonary vascular resistance (PVR) > 2 Wood units (WUs). All patients had underlying CTD. The diagnosis of concomitant ILD was confirmed by the presence of diffuse parenchymal lung disease on chest CT imaging and based on pulmonary function tests (PFTs). Other forms of PH, including chronic thromboembolic pulmonary hypertension (CTEPH), were ruled out. Patients were receiving background PAH therapy targeting the nitric oxide, endothelin, and prostacyclin pathways for at least 120 days prior to initiating sotatercept (Table 1).

Baseline hemodynamic and clinical measurements were obtained, including age, gender, type and duration of CTD, duration and treatment of PAH, and type and treatment of ILD. Additional variables assessed at baseline and follow-up included six-minute walk distance (6MWD in meters), PVR in Wood units, WHO Functional Class, N-terminal prohormone brain natriuretic peptide (NT-proBNP in pg/mL), echocardiographic measurement of estimated right ventricular systolic pressure (eRVSP in mmHg), and supplemental oxygen therapy (in liters per minute, L/min).

A follow-up period of 24 weeks was selected based on its established relevance in PH literature [4]. Notably, the STELLAR study, which led to sotatercept’s FDA approval for PAH, demonstrated significant results at 24 weeks [4].

A one-sample *t*-test was conducted for six key variables to assess whether the mean changes after 24 weeks of sotatercept treatment were statistically significant. Given the small sample size (*n* = 7), the *t*-test was chosen to compare observed sample means with hypothesized population means. Statistical significance was determined using two-tailed tests at an alpha level of 0.05. All the analyses were performed using Python 3.11. The patients and the public were not involved in the design, conduct, reporting, or dissemination plans of this research.

## 3. Results

The study included seven patients with a mean age of 57 years (range: 39–73 years), five of whom were female. All the patients had CTD-PAH with concomitant ILD. The cohort consisted of five patients with scleroderma and two with mixed connective tissue disease (MCTD), with a mean CTD duration of 2.9 years (range: 1–4 years). The patients had a mean PAH duration of 1.7 years (range: 1–3 years) and were receiving background PAH therapies targeting the nitric oxide, endothelin, and prostacyclin pathways. Of these, five patients were receiving treprostinil, while two were receiving selexipag. All the patients had nonspecific interstitial pneumonia (NSIP) as their ILD sub-type, and two were treated with anti-fibrotic therapy with nintedanib. CTD treatment included mycophenolate mofetil (5/7), hydroxychloroquine (4/7) (Table 1). The mean predicted forced vital capacity (FVC) was 62.0%, with the lowest being 55%, and the mean predicted diffusing capacity for carbon monoxide (DLCO) was 39.1%, with the lowest being 31% (Table 1).

All the patients were evaluated over a 24-week period while receiving sotatercept therapy. Sotatercept was well-tolerated, with all the patients reaching the target dose of 0.7 mg/kg and completing the 24-week study without dose interruptions or discontinuations. Headache was the most common adverse effect, reported in five of seven patients, typically within 12 h of injection and resolving with acetaminophen without interrupting therapy. Nausea occurred in four patients, and diarrhea in three; both were grade 1, self-limited, and managed conservatively. Minor epistaxis was noted in one patient, resolving spontaneously, while telangiectasia developed in one after the third dose and remained stable. No grade ≥ 3 toxicities emerged, and no patient required dose reduction or discontinued treatment because of adverse events; serial labs showed no clinically significant changes in hemoglobin, platelet count, or renal and hepatic function.

**Figure 1:**
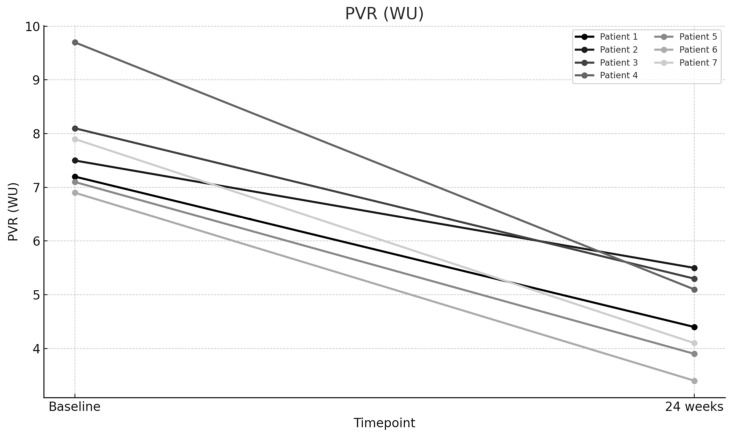
Change in pulmonary vascular resistance (PVR) from baseline to Week 24.

**Figure 2:**
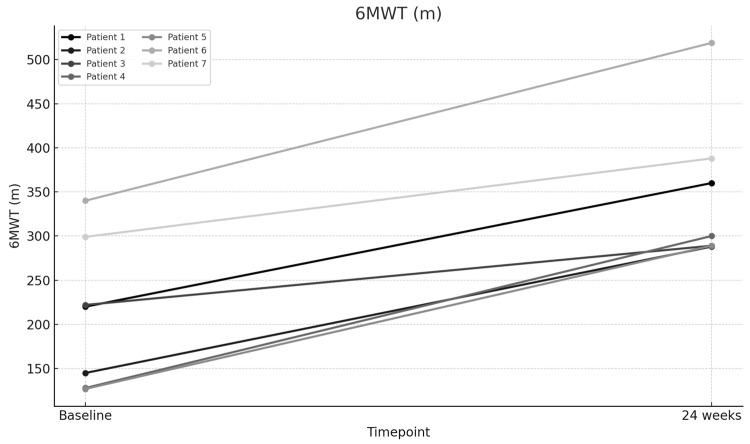
Change in six-minute walk distance (6MWD) from baseline to Week 24.

**Figure 3:**
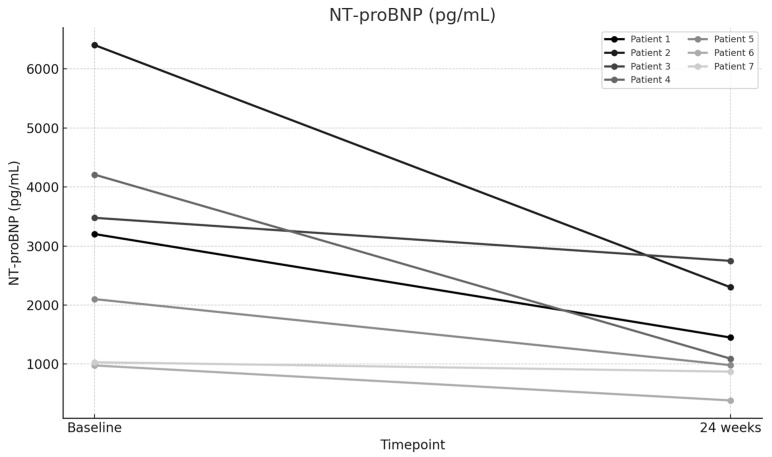
Change in N-terminal pro–B-type natriuretic peptide (NT-proBNP) from baseline to Week 24.

**Figure 4:**
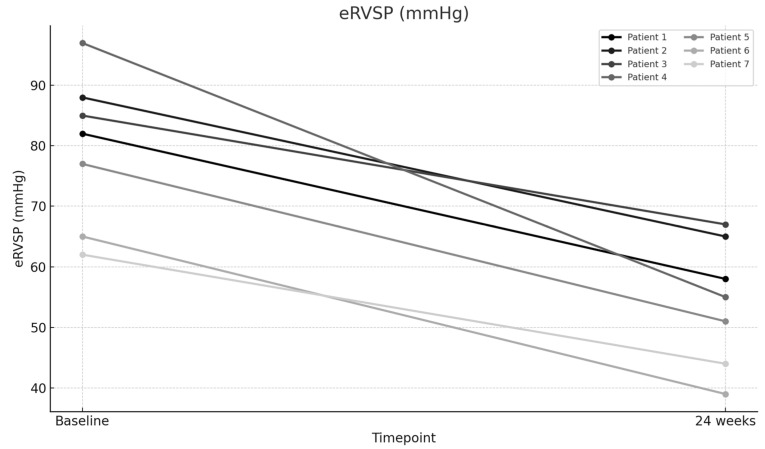
Change in estimated right ventricular systolic pressure (eRVSP) from baseline to Week 24.

The mean baseline PVR was 7.78 Wood units (WUs), which significantly improved to a mean of 4.53 WU after 24 weeks of therapy (*p* < 0.001), representing a 41.73% improvement (Figure 1). The mean 6MWD increased significantly from 211.57 m at baseline to 347.57 m at 24 weeks (*p* < 0.001), representing a 64.28% improvement (Figure 2). The mean NT-proBNP level decreased markedly from 3056.86 pg/mL at baseline to 1404.29 pg/mL at 24 weeks (*p* < 0.001), representing a 54.06% reduction (Figure 3). The mean eRVSP improved significantly from 79.43 mmHg at baseline to 54.14 mmHg at 24 weeks (*p* < 0.001) (Figure 4). Mean supplemental oxygen requirements also decreased, from 3 L/min at baseline to 1.14 L/min at 24 weeks (*p* < 0.001). WHO functional class improved in all the patients by the end of the study (Table 2 and Table 3).

These results demonstrate consistent and statistically significant improvements across multiple clinical and hemodynamic parameters.

## 4. Discussion

Patients with CTD-PAH and concomitant ILD present a unique management challenge, as they exhibit characteristics of both Group 1 PAH and Group 3 PH [2,12,13,14]. The presence of ILD complicates treatment strategies, since PAH-directed therapies, which primarily promote pulmonary vasodilation, may exacerbate V/Q mismatch, potentially worsening oxygenation [12,15,16,17]. Therefore, therapeutic decisions in this population require a careful balance between optimizing pulmonary vascular function and avoiding further impairment of gas exchange.

In addition to PDE-5i, ERAs, sGC stimulators, and prostacyclin-based therapies, sotatercept has recently emerged as a novel therapy for PAH. Sotatercept is an ASI that modulates the balance between pro- and anti-proliferative pathways in pulmonary vascular remodeling [4]. Clinical trials, including SPECTRA, PULSAR, ZENITH, HYPERION, STELLAR, and SOTERIA, have demonstrated that sotatercept increases exercise capacity, improves WHO functional class, and reduces the risk of clinical worsening in patients with PAH [4,18,19,20,21]. Additionally, these studies reported a decreased risk of death and sustained long-term benefits, reinforcing sotatercept’s durability and efficacy in PAH management [4,18,19,20,21,22].

Although sotatercept is being investigated in WHO Group 2 PH [23], there are no active studies currently evaluating its use in Group 3 PH. Previous attempts to use systemic vasodilators in Group 3 PH have yielded disappointing results, with some therapies even worsening outcomes [12,15,16]. For example, the RISE-IIP trial demonstrated that riociguat failed to improve exercise capacity and was associated with increased mortality in patients with PH-ILD [9]. Similarly, multiple studies have shown that ERAs failed to provide clinical benefit in PH-ILD, with no significant improvement in hemodynamics, functional capacity, or survival outcomes [8,24,25,26,27,28]. However, the INCREASE trial led to the FDA approval of inhaled treprostinil, marking a pivotal advancement in Group 3 PH treatment [29]. At the same time, new therapies for PH-ILD are under investigation, including a phase 3 clinical trial of inhaled seralutinib [30] and a phase 2 clinical trial of inhaled mosliciguat [31]. Despite these advancements, a critical need remains for additional therapeutic strategies to improve outcomes in PH-LD [10,16].

In this cohort of seven patients with CTD-PAH and concomitant ILD, sotatercept demonstrated significant improvements in PVR, eRVSP, 6MWD, WHO Functional Class, NT-proBNP, and supplemental oxygen requirements. Notably, these benefits were achieved without compromising oxygenation; in fact, all patients demonstrated improved oxygenation. Furthermore, sotatercept was well tolerated, with no treatment interruptions or dose reductions due to adverse effects, reinforcing its favorable safety profile in this population.

While sotatercept has demonstrated significant clinical and hemodynamic benefits in patients with CTD-PAH, its potential role in Group 3 PH, particularly in PH-ILD patients without Group 1 PAH features, remains unexplored. Given the observations in the current cohort and the limited treatment options available for PH-ILD, studies to evaluate whether sotatercept could offer a novel therapeutic approach for PH-ILD should be considered.

A key strength of this study is its real-world evaluation of sotatercept in clinical practice for patients with CTD-PAH and concomitant ILD. However, several limitations should be acknowledged. First, the small sample size restricts the generalizability of the findings, and the results should be interpreted with caution. Second, the 24-week follow-up period may not fully capture the long-term efficacy and safety of sotatercept in this population. Lastly, as a single-center study, the findings may be influenced by institution-specific practices and may not be fully representative of broader clinical settings. Multi-center, long-term studies are needed to further validate these results.

## 5. Conclusions

Sotatercept was well tolerated in patients with CTD-PAH and concomitant ILD, with no evidence of adverse respiratory effects or treatment interruptions. When added to foundational PAH therapy, sotatercept resulted in significant improvements in PVR, eRVSP, TAPSE, 6MWD, WHO Functional Class, and REVEAL 2.0 risk scores, demonstrating both hemodynamic and functional benefits in patients with Group 1 and Group 3 PH. These benefits were achieved without compromising oxygenation; in fact, all patients demonstrated improved oxygenation, further supporting its potential safety profile in this complex population. These findings suggest that sotatercept may be a promising therapeutic option as an adjunctive treatment in this patient population, and studies evaluating its effects in PH-ILD patients should be considered.

## Figures and Tables

**Table 1:** Baseline demographics and treatment characteristics of patients.

Parameter	Patient 1	Patient 2	Patient 3	Patient 4	Patient 5	Patient 6	Patient 7
Age (years)	55	42	57	71	73	39	62
Gender	F	F	M	F	M	F	F
CTD Type	Scleroderma	Scleroderma	MCTD	MCTD	Scleroderma	Scleroderma	Scleroderma
CTD Duration (years)	3	2	4	4	1	4	2
PAH Duration (years)	2	1	2	3	1	2	1
PAH Treatment	Riociguat, macitentan, selexipag	Tadalafil, macitentan, inhaled treprostinil	Tadalafil, macitentan, IV treprostinil	Tadalafil, macitentan, inhaled treprostinil	Tadalafil, macitentan, inhaled treprostinil	Tadalafil, macitentan, selexipag	Tadalafil, macitentan, inhaled treprostinil
ILD Type	NSIP	NSIP	NSIP	NSIP	NSIP	NSIP	NSIP
ILD Treatment	None	Nintedanib	Nintedanib	None	None	None	None
CTD Treatment	Mycophenolate mofetil Hydroxychloroquine	Mycophenolate mofetil	Mycophenolate mofetil Hydroxychloroquine	Hydroxychloroquine	Hydroxychloroquine	Mycophenolate mofetil	Mycophenolate mofetil
Predicted FVC (%)—Baseline	62	69	71	60	55	59	58
Predicted DLCO (%)—Baseline	39	41	33	37	49	31	44

F: female; M: male; CTD: connective tissue disease; MCTD: mixed connective tissue disease; PAH: pulmonary arterial hypertension; ILD: interstitial lung disease; NSIP: nonspecific interstitial pneumonia.

**Table 2:** Baseline and 24-week follow-up clinical and hemodynamic parameters of patients.

Parameter	Patient 1	Patient 2	Patient 3	Patient 4	Patient 5	Patient 6	Patient 7
PVR (WU)—Baseline	7.2	7.5	8.1	9.7	7.1	6.9	7.9
PVR (WU)—24 weeks	4.4	5.5	5.3	5.1	3.9	3.4	4.1
WHO Functional Class—Baseline	3	4	4	4	3	3	3
WHO Functional Class—24 weeks	2	3	3	2	1	1	1
6-Min Walk Test (m)—Baseline	220	145	222	128	127	340	299
6-Min Walk Test (m)—24 weeks	360	288	289	300	289	519	388
NT-proBNP (pg/mL)—Baseline	3202	6402	3477	4208	2100	977	1032
NT-proBNP (pg/mL)—24 weeks	1450	2301	2748	1092	982	385	872
eRVSP (mmHg)—Baseline	82	88	85	97	77	65	62
eRVSP (mmHg)—24 weeks	58	65	67	55	51	39	44
Supplemental O_2_ Therapy (L/min)—Baseline	4	6	4	3	2	0	2
Supplemental O_2_ Therapy (L/min)—24 weeks	2	4	2	0	0	0	0

PVR: pulmonary vascular resistance; WUs: Wood units; WHO: World Health Organization; 6MWT: Six-Minute Walk Test; m: meters; NT-proBNP: N-terminal prohormone brain natriuretic peptide; pg/mL: picograms per milliliter; eRVSP: estimated right ventricular systolic pressure; mmHg: millimeters of mercury; O_2_ Therapy: supplemental oxygen therapy; L/min: liters per minute.

**Table 3:** Summary of paired *t*-test results for clinical parameters after 24 weeks of treatment.

Parameter	Baseline Mean	Mean After 24 Weeks	Difference	Percentage Change (%)	*T* Score	*p* Value
PVR (WU)	7.77	4.53	3.24	41.7	10.308	0.000049
6MWD (m)	211.57	347.57	136	64.28	8.459	0.00015
NT-proBNP (pg/mL)	3056.86	1404.29	1652.57	54.06	3.013	0.02362
eRVSP (mmHg)	79.43	54.14	25.29	31.84	8.262	0.00017
Supplemental O_2_ (L/min)	3	1.14	1.86	62	5.461	0.00157

PVR: pulmonary vascular resistance; WU: Wood units; 6MWD: six-minute walk distance; m: meters; NT-proBNP: N-terminal pro-brain natriuretic peptide; pg/mL: picograms per milliliter; eRVSP: estimated right ventricular systolic pressure; mmHg: millimeters of mercury; O_2_ Therapy: supplemental oxygen therapy; L/min: liters per minute.

## Data Availability

The original contributions presented in this study are included in the article. Further inquiries can be directed to the corresponding author.

## Abbreviations

The following abbreviations are used in this manuscript:

ASI	Activin Signaling Inhibitor
CTD	Connective Tissue Disease
CTD-PAH	Connective Tissue Disease-Associated Pulmonary Arterial Hypertension
DLCO	Diffusing Capacity for Carbon Monoxide
eRVSP	Estimated Right Ventricular Systolic Pressure
ERA	Endothelin Receptor Antagonist
FDA	Food and Drug Administration
FVC	Forced Vital Capacity
ILD	Interstitial Lung Disease
MCTD	Mixed Connective Tissue Disease
mPAP	Mean Pulmonary Artery Pressure
NSIP	Nonspecific Interstitial Pneumonia
NT-proBNP	N-terminal Prohormone Brain Natriuretic Peptide
PAH	Pulmonary Arterial Hypertension
PCWP	Pulmonary Capillary Wedge Pressure
PDE-5i	Phosphodiesterase-5 Inhibitor
PH	Pulmonary Hypertension
PH-ILD	Pulmonary Hypertension Associated with Interstitial Lung Disease
PFT	Pulmonary Function Test
PVR	Pulmonary Vascular Resistance
RHC	Right Heart Catheterization
RISE-IIP	Riociguat for Idiopathic Interstitial Pneumonia
RCT	Randomized Controlled Trial
sGC	Soluble Guanylate Cyclase
TAPSE	Tricuspid Annular Plane Systolic Excursion
V/Q	Ventilation-Perfusion
WHO	World Health Organization
WUs	Wood Units
6MWD	Six-Minute Walk Distance
